# Everybody's Page

**Published:** 1889-07-13

**Authors:** 


### Everybody's Page.

Inhalations of spirit of chloroform are said to cut short attacks of acute rheumatism.

The disagreeable after-taste of salicylic acid and salicylate of soda can be avoided by putting a small quantity of common salt on the tongue before administering them.

A Russian doctor speaks enthusiastically of what he calls "urtication"—that is, pricking with a bunch of fresh nettles —as a cure for anæsthesia, neuralgia, and numerous other diseases. It has long been in use among the Russian peasantry.

Drs. Rossi and Agostini have recently made some experiments with bromide of potassium, which prove that its action is to paralyse the nervous centre, causing diminished sensibility, torpor, and finally death. It has a direct action on the brain and spinal cord.

Now that consumption is gradually coming to be regarded as a contagious disease, people are beginning to ask how it it is to be prevented. Dr. Cornet, of Berlin, strongly advises that phthisical patients should use their own spoons, glasses, etc., and should avoid kissing their friends. If death occurs, the room occupied by the dead person should be rubbed with new bread, and all furniture, bedding, etc., should be carefully disinfected.

The *Scots Observer* says some harsh things about specialism, while admitting that "a wise, an unexaggerated specialism results in scientific progress." It says: "In health, to keep a homœopathist in constant attendance, and for sickness, to hold in reserve a regular medical man, is an amusement harmless in itself, less expensive than keeping a racing stable, and not dangerously exciting. But to have a special doctor for every organ is a somewhat risky amusement which any metropolis puts in the way of hypochondriacs."

In the Amsterdam Hospital it is found that rheumatic fever often attacks patients in batches, and the attendants there speak of "rheumatism days" when this occurs. Not long ago a patient who was recovering from typhoid was suddenly seized with acute rheumatism, without having shown any premonitory symptoms. The only cause that could be assigned for the attack was that her bed had been placed between those of two rheumatic patients. The inference is that the rheumatism was the result of infection from the other patients. If this be true, it will be necessary to put rheumatism in the already long list of infectious diseases.

Tea came into use almost by accident. Some Buddhist priests, going on a missionary expedition from Northern India to China, took with them the dried leaves and also some cuttings of an indigenous shrub, which was said to have the power of correcting any injurious properties in the brackish water they might meet with on the way. The decoction thus made pleased the missionaries so well that they continued, as a matter of taste, to drink it after they had reached China, and introduced it to their converts. They also set about planting the precious shrub, and, although it did not thrive so well in China as in its native Assam, becoming smaller both in stem and leaf, it was so well liked that it soon formed the foundation of the favourite beverage of all China. Thence it was brought to Europe, to be drunk and desired by Englishmen of every degree. And it is only of late years that Assam tea has come into the European market, to be looked upon rather suspiciously as the rival of its own degenerated Chinese daughter.

As lately as 1748, a London patent medicine manufacturer was advertising belts for the cure of leprosy at the price of 6s. each.

Short-sightedness descends from parent to child in diagonal succession; that is, sons inherit it most frequently from their mothers, and daughters from their fathers. The average of such cross-transmissions is about 80 per cent., direct transmission from father to son and *vice versâ* being much less frequent.

Sponge culture promises to be a thriving industry on the Austrian seaboard. Three years ago an Austrian scientist sowed small parts of living sponges on a warm submarine soil. The process cost £11, and he is now rewarded by a harvest of 4,000 sponges. The Government intends to take up the industry.

Dr. John Morris, an American physician, thinks that the family doctor should be consulted in the education of children, as if he has any appreciation of the value of heredity he will be able to suggest the training and future profession that will best correct any taint of blood, and put least strain on the weakest parts of body and mind.

Japan wax is a curious product obtained from a tree common in the East Indies, and called in Japan "haje." The tree begins to bear fruit when about five years old, and goes on increasing in produce steadily. When about fifty years old it yields often 350 pounds of berries, from which 70 pounds of wax can be obtained. The wax lies between the seed and the skin of the berry.

In our search for a cure for hydrophobia, why does no one think of seeking the M'Govan family, in county Cavan, who have kept the secret of one for nearly 300 years. It is, of course, impossible to tell what the cure is, for it is confined to the breast of one man. The father, on his death-bed, confides it to his son, and so it goes down. All that is generally known is that the patient is treated with a broth of herbs, which is said to be far from appetising. These herbs are gathered in the early dawn, but whether some mystic virtue lies in the hour they are culled, or whether it is done only to keep off prying eyes, no one can tell. The fact remains that many people who have been bitten by rabid dogs, and not merely dread hydrophobia, but are in foaming convulsions from it, have been cured by the M'Govan's treatment. Myth and exaggeration may have gathered around it, but even so, is it not worthy to engage the attention of our scientists?

Dr. J. Warren Achorn writes an indignant letter to the American *Medical Record* describing an examination that recently took place for the appointments in Bellevue Hospital, New York. One examiner confessed that he had asked catch questions, which no one who had not been "put up" to them before could answer, "just for the fun of it." And when they failed he ploughed them—doubtless "just for the fun of it." Another solemnly plied the candidates with questions which might win them the appointment, but certainly would not prove that they could treat a simple headache or a cut finger if they did obtain it. Similar questions set to nurses have recently been quoted in our columns. Is it not time that examination questions should cease to emulate the classic example: "Who dragged whom, how many times, round the walls of where?" After all, medicine and nursing are serious professions, not Christy minstrel entertainments, and the applause and rewards should not go to those who can answer useless conundrums the most successfully.

